# The efficacy of loco-regional ropivacaine analgesia via intercostal catheters after lung resection: a randomized, double-blind, placebo-controlled, superiority study

**DOI:** 10.1093/ejcts/ezae342

**Published:** 2024-10-01

**Authors:** Aljaz Hojski, Monica Krämer, Paulius Gecas, Zeljko Djakovic, Nikolay Tsvetkov, Makhmudbek Mallaev, Daniel Bolliger, Andreas Lampart, Didier Lardinois

**Affiliations:** Department of Thoracic Surgery, University Hospital Basel, University of Basel, Basel, Switzerland; Department of Thoracic Surgery, University Hospital Basel, University of Basel, Basel, Switzerland; Department of Thoracic Surgery, University Hospital Basel, University of Basel, Basel, Switzerland; Department of Thoracic Surgery, University Hospital Basel, University of Basel, Basel, Switzerland; Department of Thoracic Surgery, University Hospital Basel, University of Basel, Basel, Switzerland; Department of Thoracic Surgery, University Hospital Basel, University of Basel, Basel, Switzerland; Clinic for Anesthesia, Intermediate Care, Prehospital Emergency Medicine and Pain Therapy, University Hospital Basel, University of Basel, Basel, Switzerland; Clinic for Anesthesia, Intermediate Care, Prehospital Emergency Medicine and Pain Therapy, University Hospital Basel, University of Basel, Basel, Switzerland; Department of Thoracic Surgery, University Hospital Basel, University of Basel, Basel, Switzerland

**Keywords:** Intercostal catheter, Minimally invasive lung surgery, Video-assisted thoracoscopic surgery, Regional anaesthesia

## Abstract

**OBJECTIVES:**

Postoperative pain remains a burden for patients after minimally invasive anatomic lung resection. Current guidelines recommend the intraoperative placement of intercostal catheters to promote faster recovery. This trial aimed to determine the analgesic efficacy of continuous loco-regional ropivacaine application via intercostal catheter and establish this method as a possible standard of care.

**METHODS:**

Between December 2021 and October 2023, patients were randomly assigned to receive ropivacaine 0.2% or a placebo through an intercostal catheter with a flow rate of 6–8 ml/h for 72 h after surgery. Patients were undergoing anatomic VATS lung resection under general anaesthesia for confirmed or suspected stage I lung cancer (UICC, 8th edition). The sample size was calculated to assess a difference in numerical rating scale associated with pain reduction of 1.5 points.

**RESULTS:**

Fourteen patients were included in the ropivacaine group, whereas the placebo group comprised 18 participants. Patient characteristics and preoperative pain scores were similar in both groups. There was no statistically significant difference in postoperative pain scores and morphine consumption between the 2 groups. The mean numerical rating scale when coughing during the first 24 h postoperatively was 4.9 (SD: 2.2) in the ropivacaine group and 4.3 (SD: 2.4); *P* = 0.47 in the placebo group. We were unable to determine any effect of administered ropivacaine on the postoperative pulmonary function (FEV1, PEF).

**CONCLUSIONS:**

Our preliminary results suggest that continuous loco-regional ropivacaine administration via surgically placed intercostal catheter has no positive effect on postoperative pain scores or morphine requirements.

**CLINICAL REGISTRATION NUMBER:**

NCT04939545

## Graphical Abstract

**Figure:**
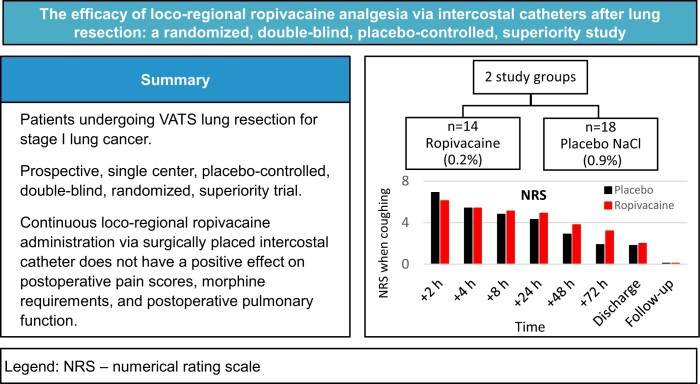

## INTRODUCTION

Video-assisted thoracoscopic surgery (VATS) is considered a minimally invasive procedure, though still associated with relevant acute perioperative and postoperative pain [1]. Preoperatively inserted thoracic epidural catheters for analgesia (TEA) might still be considered the gold standard for pain control after major thoracic surgery [1]. However, their use is hampered by the occurrence of possible serious side effects, especially when taking anticoagulants, suffering from infectious diseases or encountering difficult anatomical conditions. In addition, TEA might restrict early patient mobilization and require the placement of an urethral catheter. Finally, hypotension is commonly associated with TEA placement and might hamper the intended early discharge according to the Enhanced Recovery after Surgery (ERAS) guidelines [2]. Developments in the field of perioperative care increasingly favour pain management tailored to the patient [3]. Direct intercostal nerve blockage is an established regional analgesic technique, avoiding the above-discussed disadvantages of TEA, which is increasingly used for early pain management after minimally invasive thoracic surgery [1–3]. The intercostal catheter (ICC) is placed by the operating surgeon and presumably blocks 2–3 adjacent intercostal nerves. It should be used in a multimodal protocol including regional anaesthesia plus oral or/and intravenous analgesia [4–6]. In contrast to the analgesic effect of local anaesthetic (LA) via the ICC, its effects on early postoperative pulmonary function have not yet been sufficiently investigated [3, 6, 7].

The primary objective of this study was to examine the analgesic effectiveness of continuous loco-regional analgesic application of ropivacaine through a surgically placed ICC. Further, we sought to correctly establish this method as a possible standard of care in postoperative analgesia after VATS anatomical lung resection.

## PATIENTS AND METHODS

In this prospective, single-centre, placebo-controlled, double-blind and randomized superiority trial, we evaluated the efficacy of ICC-administered pain treatment.

After approval of the Ethics Review Board: EKNZ (Ethikkommission Nordwest- und Zentralschweiz) BASEC 2021-00922, 2021-06-18, the study was registered at ClinicalTrials.gov, NCT04939545 and conducted between December 2021 and October 2023. Patients provided informed written consent for their inclusion in the study and publication of their study data.

### Inclusion criteria

Patients aged ≥ 18 years undergoing an anatomic VATS lung resection under general anaesthesia for confirmed or suspected stage I lung cancer (UICC, 8th edition), and who had a physical status classification I to III according to the American Society of Anesthesiologists (ASA).

### Exclusion criteria

Patients with preoperative numerical rating scale (NRS) while coughing >0, previous ipsilateral thoracotomy, sternotomy, abdominal or contralateral thoracic surgery up to 6 months preoperatively were excluded. Patients with contraindications to the drug class, self-administration of opioids, steroid therapy lasting more than 2 weeks prior to surgery or daily pain therapy were not included in the study either. Pregnant or breast-feeding women, patients with congestive heart failure and/or liver insufficiency, and patients with a known inability to follow the procedures of the study were excluded from the study. Further, patients who participated in another study within 30 days prior to being included in our study were also excluded.

### Methods of minimizing bias

Participants were randomly assigned to the ropivacaine and placebo groups once we obtained written informed consent from them. A designated statistician, otherwise not involved in this study, created a randomization list with a 1:1 ratio for the block randomization. All investigators, care providers, the data analyst and trial participants were blinded. In addition, an ICC was placed intraoperatively in all patients to complement these methods of minimizing bias.

### Intervention and pain management

The patients’ baseline pain data and pulmonary function were obtained 1 day prior to surgery. Surgery was standardized using the 3-port technique with an Alexis^®^ soft tissue retractor and 2 caudal plastic ports [4]. Patients underwent anatomical resections of the pulmonary parenchyma and mediastinal lymphadenectomies. After the resection was completed, the surgeon thoracoscopically placed an ICC at the level of the mini-thoracotomy [4th or 5th intercostal spaces (ICS)], as previously described, using a bolus of 20 ml IMP (investigational medicinal product) according to randomization for hydro-dissection [4, 5] and inserted a 20Ch Portex^®^ thoracic catheter through the 11.5 cm camera port (Medtronic, Ireland). All catheters were inserted by the same surgeon. Either ropivacaine at a concentration of 2 mg/ml [ROPIvacain Fresenius 0.2%, Fresenius Kabi (Schweiz) AG, Kriens, Switzerland] or the placebo (NaCl 0.9%, B. Braun, Medical AG, Sempach, Switzerland) was administered via the ICC, dosed with an elastomer pump (AutoSelector, Acemedical, Korea, CE0120) until 72 h after surgery. We started the application of the investigational medicinal product at a flow rate of 6 ml/h and increased it to 8 ml/h when the NRS exceeded 72 h after skin closure. If there was no air leakage and fluid secretion was below 200 ml within 24 h, the chest tube was removed. All patients were treated for pain according to a standardized multimodal analgesia protocol including NSAIDs (nonsteroidal anti-inflammatory drugs), metamizole (dipyrone) and paracetamol (acetaminophen). The use of postoperative oral analgesics and morphine was documented as part of the postoperative follow-up.

### Spirometry measurements

The investigators performed bedside spirometry tests with the patient in a sitting position, using a handheld spirometry system (AioCare™, HealthUp S.A., Poland, CE 2274) to assess the analgesic impact on early postoperative pulmonary function. The highest values out of 3 measurements were documented. Forced expiratory volume in the first second (FEV1) and peak expiratory flow (PEF) were measured preoperatively and 24, 48 and 72 h postoperatively, as well as 1 day before discharge and 6 months after surgery. The interpretation of the obtained spirometry data was done according to the equations from GLI-2017 (Global Lung Initiative of the European Respiratory Society) [8, 9] for FEV1 and ERS/ECCS 1993 [10] for PEF.

### Clinical outcomes

The primary outcome variable, superior postoperative analgesia when the patient is coughing, was assessed through the NRS and morphine consumption 2, 4, 8, 24, 48 and 72 h after skin closure by comparing the 2 groups.

Secondary outcome variables included pain scores at rest assessed through the NRS and pulmonary function. Other outcomes of interest were nausea, vomiting, and bowel movement assessed 2, 4, 8, 24, 48 and 72 h after skin closure. In addition, the time to first defecation and length of hospital stay (defined as time from surgery until discharge from our hospital) were included as secondary outcome variables as well.

Patients were mobilized as early as the first postoperative day and performed daily incentive spirometry and physiotherapy. They stayed in hospital for an average of 7 days, and were all included in an oncological follow-up. A study-specific follow-up was scheduled 6 months (±42 days) postoperatively, encompassing pain history and pulmonary function tests.

### Statistical methods

The sample size was calculated to detect an NRS difference associated with a pain reduction of 1.5 points using a paired *t*-test and a within-subject variability of 1.7 pain scores. The descriptive statistics included the frequency and proportion, median and interquartile range (IQR), or mean and standard deviation (SD). The corresponding *P*-values were derived from significance tests such as the Chi-squared test, Fisher’s exact test, Wilcoxon rank-sum test or *t*-test.

The primary endpoint ‘NRS when coughing’ was analysed on the original scale as verified, both by quantile plots and plots representing standardised residuals versus fitted values. To calculate the resulting effect sizes for each timepoint separately, linear mixed-effects models were performed with a nested design, namely study groups nested in time. No potential significant non-linearities or interactions were detected in the preliminary analysis. The linear mixed-effect models were adjusted for the total amount of morphine administered, as well as for age, gender, and body mass index (BMI). Consequently, the difference in mean values, 95% confidence intervals (CIs) and *P*-values were indicated. A *P*-value <0.05 was considered significant. All analyses were performed with the statistical program R version 4.2.2. [11].

## RESULTS

While 14 patients were included in the ropivacaine group, there were 18 in the placebo group. The patients’ characteristics are presented in Table 1. A total of 15 lobectomies and 17 segmentectomies were performed, with more lobectomies in the placebo group (*n* = 10) and one more segmentectomy in the ropivacaine group (*n* = 9). There were no differences in the relevant intra- and postoperative parameters (duration of surgery and anaesthesia medications) and in other relevant hospitalization data between both groups (Table 1). The median time with the chest tube for all patients was 41.4 [19.1, 73.8] h. Patients in the ropivacaine group received a mean dose of 879 (SD: 191.2) mg ropivacaine over 3 days. In both groups, the change in flow rates from 6 ml/h to 8 ml/h occurred to the same extent (Table 1).

**Table 1:** Baseline, operation and hospitalization-related characteristics in all patients by study group, where appropriate, reported as mean values with standard deviation (SD), otherwise as median (IQR)

	Placebo (*n* = 18)	Ropivacaine (*n* = 14)	*P*/test method
Patient characteristics			
Female (%)	10 (55.6)	9 (64.3)	0.89/Chi^2^test
Age (years) at operation	69.7 (9.3)	72.9 (8.5)	0.32/*t*-test
BMI (kg/m^2^)	27.4 (5.2)	27.3 (5)	0.95/*t*-test
Operation Duration (min)	230.5 (65.3)	239.6 (52.9)	0.67/*t*-test
Intraoperative analgesia			
Fentanyl total (µg)	480.6 (156.4)	457.1 (137.1)	0.66/*t*-test
Fentanyl (µg/kg)	6.3 (2)	6.1 (1.9)	0.80/*t*-test
Remifentanil (µg/kg)	1791.7 (484.7)	1698.1 (536.4)	0.61/*t*-test
Metamizole (dipyrone) 1 g			1/Fisher’s exact test
Yes	16	13	
No	2	1	
Hospitalization IMP^a^ application			
Change of flow rate			0.45/Fisher’s exact test
Yes (%)	7 (38.9)	3 (21.4)	
No (%)	11 (61.1)	11 (78.6)	
Duration 6 ml/h (h)	45.7 (18.6–71.9)	66.7 (65.6–75.1)	0.12/Ranksum test
Duration 8 ml/h (h)	54.8 (18)	29.9 (15.1)	0.071/*t*-test
Chest tube duration (h)	44.2 (28–83.1)	21.1 (17.9–61.1)	0.071/Ranksum-test
Length of hospitalization (days)	7 (6–8)	5.5 (5–7.8)	0.26/Ranksum-test

a Investigational medicinal product.

**Figure 1:**
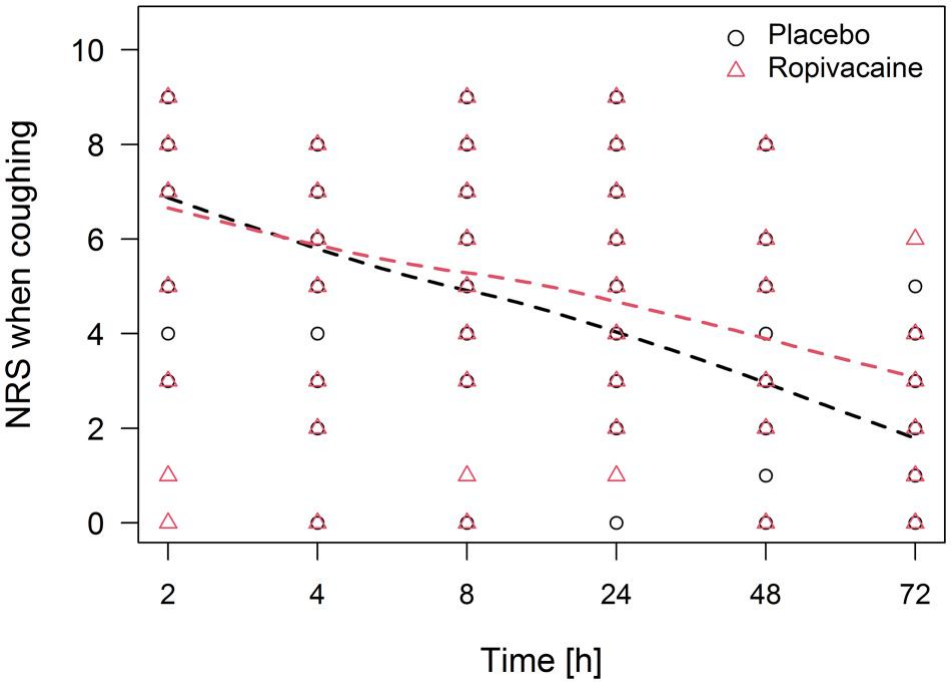
Postoperative pain scores when coughing after minimally invasive anatomical lung resection with or without loco-regional analgesia through intercostal catheter.

**Figure 2:**
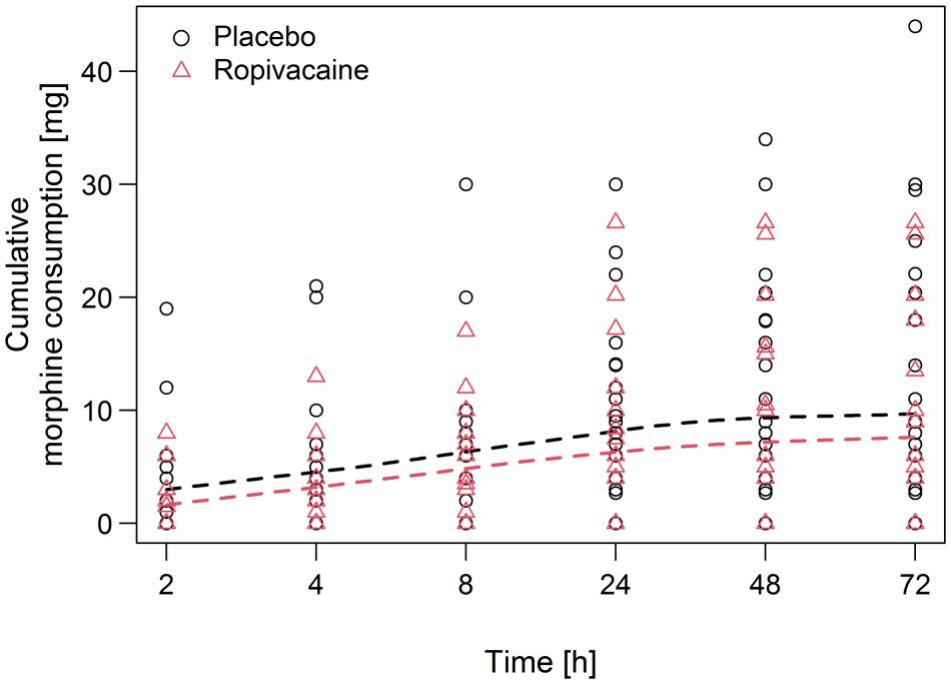
Postoperative cumulative morphine consumption at respective times.

A comparison between the 2 groups revealed no statistically significant difference in postoperative pain scores and morphine consumption (Table 2, Figs 1 and 2). The administered amounts of additional analgesics were similar in both groups. The NRS score when coughing in the first 24 hours postoperatively was 4.9 (SD: 2.2) in the ropivacaine group and 4.3 (SD: 2.4) in the placebo group; *P* = 0.47. Morphine consumption at 24 h after surgery was similar in the ropivacaine and in the placebo group [9.2 (SD: 7.6) vs 10.9 (SD: 8.1) mg; *P* = 0.55]. The NRS scores when coughing after the first 72 h postoperatively tended to be higher in the ropivacaine than in the placebo group [3.2 (SD: 1.7) vs 1.9 (SD: 1.9); *P* = 0.068], but there was no difference in cumulative morphine consumption between the ropivacaine and the placebo group [11.1 (SD: 9) mg vs 14.3 (SD: 12) mg; *P* = 0.42]. The average pain scores at rest were below NRS 2 48 h after surgery and below NRS 1 72 h after the operation. The chest drains were removed more than 20 h earlier in the ropivacaine group (Table 1). For most patients, the average pain score when coughing was NRS 2 or less at discharge. Two patients reported pain (NRS ≤ 3) at rest, 4 patients reported varying degrees of pain when coughing 6 months after surgery, with no difference between the groups (Table 2, Fig. 1). None of them required any pain medications. We observed no major side effects of morphine. Hospitalization was uneventful for all the patients in the absence of morbidity or mortality.

**Table 2:** Summary statistics of pain and morphine consumption across all patients and by study group during hospitalization and pain in follow-up, reported as mean values with standard deviation (SD) or median (IQR)

	Placebo (*n* = 18)	Ropivacaine (*n* = 14)	*P*/*t*-test
After 2 h			
NRS	5.7 (2.1)	4.6 (2.7)	0.24
NRS (coughing)	6.9 (1.9)	6.1 (2.9)	0.39
morphine (mg)	3.8 (4.8)	1.8 (2.5)	0.18
After 4 h			
NRS	3.8 (2.2)	3 (2.3)	0.33
NRS (coughing)	5.4 (2.4)	5.4 (2.3)	0.97
Morphine (mg)	6.1 (5.9)	3.6 (3.7)	0.17
After 8 h			
NRS	2.3 (2.2)	3.3 (2.6)	0.27
NRS (coughing)	4.8 (2.5)	5.1 (2.9)	0.75
Morphine (mg)	7.6 (7.3)	5.5 (5)	0.39
After 24 h			
NRS	2.1 (2.1)	2.6 (2.3)	0.50
NRS (coughing)	4.3 (2.4)	4.9 (2.2)	0.47
Morphine (mg)	10.9 (8.1)	9.2 (7.6)	0.55
After 48 h			
NRS	1.3 (1.6)	1.9 (2.2)	0.39
NRS (coughing)	2.9 (2.5)	3.8 (2.2)	0.33
Morphine (mg)	12.6 (9.7)	10.8 (8.7)	0.58
After 72 h			
NRS	0.7 (1)	0.9 (1.4)	0.65
NRS (coughing)	1.9 (1.9)	3.2 (1.7)	0.068
Morphine (mg)	14.3 (12)	11.1 (9)	0.42
Discharge—1 day			
NRS	0.7 (1.1)	0.3 (0.6)	0.25
NRS (coughing)	1.8 (1.8)	2 (1.4)	0.77
Discharge			
Morphine (mg)	15.4 (13.3)	11.8 (9.6)	0.41
Follow-up 180 days			
NRS	0 (0, 0)	0 (0, 0)	0.096 (Ranksum)
NRS (coughing)	0 (0, 0)	0 (0, 0)	0.63 (Ranksum)

**Figure 3:**
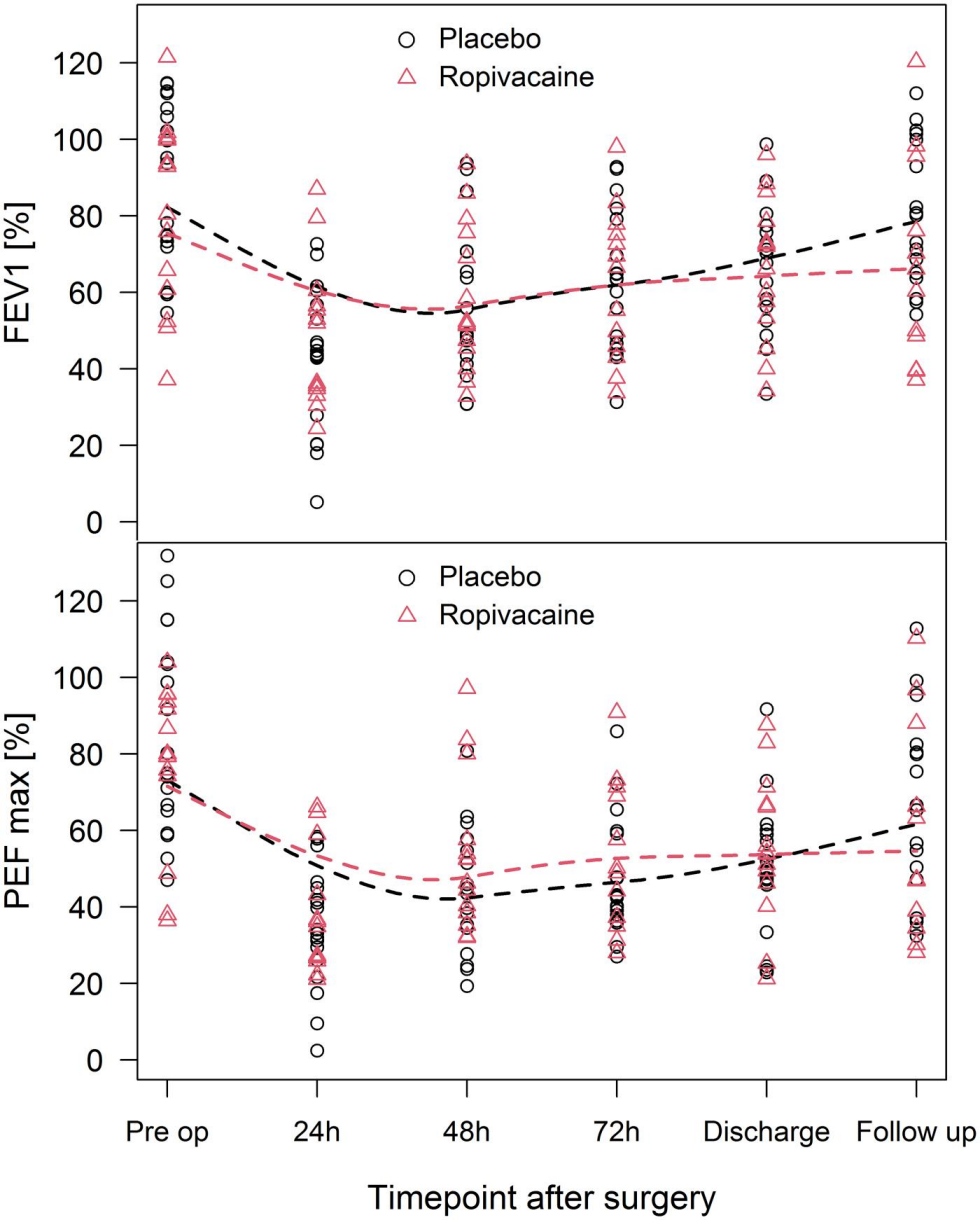
Postoperative pulmonary function values, the forced expiratory volume in first second and peak expiratory flow max in % of target.

In addition, administration of ropivacaine was found to have no effect on the postoperative pulmonary function as assessed by FEV1, and PEF (Table 3, Fig. 3).

**Table 3:** Summary of pulmonary function (% of target) by study group, reported as mean values with standard deviation (SD)

Pulmonary function (% target)	Placebo (*n* = 18)	Ropivacaine (*n* = 14)	*P*/*t*-test
Preoperative			
FEV1	89.2 (20.8)	81.0 (24.5)	0.31
PEF	85.3 (26.1)	77.1 (21.5)	0.35
After 24 h			
FEV1	43.2 (18.6)	48.1 (18.7)	0.47
PEF	34.7 (15.7)	37.7 (15.3)	0.59
After 48 h			
FEV1	57.9 (19.9)	58.6 (19.1)	0.93
PEF	44 (16.4)	53.3 (20.2)	0.17
After 72 h			
FEV1	63 (18.2)	62.2 (19.5)	0.91
PEF	47.4 (15.4)	52.9 (18.8)	0.38
Discharge—1 day			
FEV1	65.5 (16.7)	66 (18.6)	0.93
PEF	49.9 (17.2)	55.1 (19.1)	0.42
Follow-up 180 days			
FEV1	80.5 (18.7)	66.8 (26.6)	0.11
PEF	65.1 (24.5)	57.4 (27.6)	0.44

## DISCUSSION

The main objective of this trial was to evaluate the efficacy of continuous loco-regional ropivacaine application via a surgically placed ICC as an adjunct to standard treatment in perioperative pain management after minimally invasive anatomic lung resection and to establish this method as a possible standard of care. Our results suggested that ropivacaine administered this way has no beneficial effect on postoperative pain, morphine consumption or other relevant outcomes, including pulmonary function.

Even with minimally invasive surgery, post-surgical pain and opiate usage should be minimized, as they both unfavourably influence recovery and outcome [3]. Regional analgesia techniques are recommended and widely used, but none has yet been established as a clear substitute for TEA [2]. ICCs are placed under endoscopic control at the end of the surgery between the parietal pleura and the endo-thoracic fascia at the level of the surgical incision [4]. There is no consensus on the optimal intercostal space (ICS) for the catheter placement, timing of insertion, at the beginning or after surgery, and the administration of analgesics, whether continuous or as boluses [4, 5]. Prolonged regional thoracic analgesia via ICC after minimally invasive anatomical lung resection was associated with less postoperative pain, easier placement compared to TEA, faster recovery, shorter duration of chest tube placement and hospitalization [1, 3–5]. Adverse effects during placement and application of analgesics by the ICC are non-existent or rare [1, 3]. Continuously infused LA over a surgically placed ICC more likely blocks only one ICS and is initially not as potent as surgically performed intercostal nerve blocks (ICNB) or paravertebral blocks performed under ultrasound guidance, mostly by anaesthesiologists [3, 4].

It remains unclear why ropivacaine via ICC did not result in pain reduction in this study. We suspected that the main pain occurred at the site of the utility incision, where the manipulation was greatest, so we placed the catheter in the 4th or 5th ICS [4, 5]. Some researchers suggested that the chest tube and the pressure directly applied on the ribs through camera movements are the main cause of pain [4]. Despite the earlier removal of the chest drain in the Ropivacaine group (Table 1), pain scores remained higher in this group. Of note, our findings contradict the results of a comparable study [4], which may be due to the facts that in this study the ICNBs were administered over several ICSs (3rd−8th), morphine was injected at the end of anaesthesia and the postoperative analgesic regime included gabapentin.

This raises the question of whether ICCs are necessary at all, as proposed by other researchers [7]. Since the TEA is normally placed at the beginning of the anaesthesia to prevent the first pain stimulus in the brain, it may be more appropriate to administer the LA at the beginning of the procedure via several ICNBs. According to the relevant guideline [2], the ICCs are usually inserted at the end of surgery in order to not disrupt the procedure [4]. We suspect that the ICC placement at the beginning of surgery might have resulted in more beneficial effects in our study [3].

A recent meta-analysis suggested an administration of LA doses about 2–3 times higher than in our study [3]. However, a study by Wildgaard *et al.* used continuously administered bupivacaine at a comparable dosage as in our study and reported a low pain intensity [4], which agrees with our findings. An alternative to continuous administration is the administration of LA as a bolus [5], for which, however, the staff must be trained and always present. In addition, fluctuation in LA concentrations might result in higher pain scores. Liposomal bupivacaine or other techniques (cryoanalgesia) could not achieve appropriate results and are therefore not recommended as standard treatment at the moment [3].

As in other studies, pain decreased to below NRS 4 48 h after surgery, suggesting that ICNBs may provide adequate postoperative analgesia in terms of duration of local anaesthesia [3, 4]. Pain scores in our study and their change over time are comparable to those in several similar studies [3, 4]. It is noteworthy to mention that a recently published study conducted at our hospital with a comparable group of patients and very similar anaesthesia protocol reported higher pain scores at rest and a morphine requirement about twice as high in patients after simple VATS procedures [12]. Participants of the previous study had access to morphine on demand via an intravenous pump. This finding could indicate that postoperative pain cannot be sufficiently alleviated with morphine alone [3]. Of note, patients in our previous study were younger and mentally less prepared for the operation, e.g. a potentially curative oncological surgery. They, therefore, might not have accepted postoperative pain as well as the patients in the present study. This might be supported by the fact that some patients of the former study were still in pain at the follow-up visit 6 months after surgery [12]. In the present study, some patients also reported persistent pain, which is a further indication that other, nonsurgical factors could also influence postoperative pain perception. Finally, another recently published study with a similar study population, reported pain scores comparable to the present finding; however, morphine requirements were about 6–7 times higher [13]. Evidence suggests that patient characteristics (age, background, etc.) have a significant influence on the perception of postoperative pain [3]. Findings from studies investigating and evaluating pain after thoracic surgery might, therefore, be relevantly affected by the patient’s preoperative condition [3].

Postoperative pulmonary function is one of the most important functional outcomes after lung resection [6] and provides information on whether a patient’s treatment was successful [7]. In our study, pulmonary function before surgery was comparable in both study groups. There was also no difference postoperatively, proposing that continuous loco-regional ropivacaine administration does not improve it. A recently published study showed no significant difference in postoperative outcomes between segmentectomy and lobectomy [14]. Further research is needed to find parameters that make a positive contribution to the recovery of the pulmonary function [3, 4]. Also, alternative combinations of medications for the regional anaesthesia should be studied further [1]. Local anaesthesia started at the beginning of the procedure and covering several intercostal spaces might be worth considering.

### Limitations

Although the sample size of the study was determined preoperatively and calculated to answer our clinical question, we consider the main limitation of our study to be the small number of participants included. Furthermore, some elastomeric pumps had to be replaced due to malfunction, which resulted in unbalanced study groups. The study was conducted in an intention-to-treat setting. Other unrecognized confounders could play a role as well, one being selection bias, as patients had to fulfil the inclusion criteria and agree to participate. The negative outcome can also be attributed to several factors, namely the location of the ICC placement, timing and type of the LA application and its dosage.

## CONCLUSIONS

Our results suggest that the analgesic efficacy of continuous loco-regionally applied ropivacaine administered through a surgically placed intercostal catheter after surgery does not have a positive effect on postoperative pain scores, morphine requirements or postoperative pulmonary function.

## Data Availability

The data underlying this article will be shared on reasonable request to the corresponding author.

## Notes

Presented and Awarded with the Grillo Prize at the 32nd European Conference on General Thoracic Surgery, Barcelona, Spain, 26–28 May 2024.

## ABBREVIATIONS

CI: confidence interval
FEV1: forced expiratory volume in first second
ICC: intercostal catheter
ICS: intercostal space
ICNB: intercostal nerve blocks
IMP: investigational medicinal product
IQR: interquartile range
LA: local anaesthesia
NRS: numerical rating scale
PEF: peak expiratory flow
TEA: thoracic epidural catheters for analgesia
VATS: Video-assisted thoracoscopic surgery
